# Therapeutic Potential of *Rhododendron arboreum* Polysaccharides in an Animal Model of Lipopolysaccharide-Inflicted Oxidative Stress and Systemic Inflammation

**DOI:** 10.3390/molecules25246045

**Published:** 2020-12-21

**Authors:** Ajaz Ahmad, Adil Farooq Wali, Muneeb U. Rehman, Andleeb Khan, Mohammad Raish, Mohsin Kazi, Osamah Alnemer, Padma G. M. Rao

**Affiliations:** 1 Department of Clinical Pharmacy, College of Pharmacy, King Saud University, Riyadh 11451, Saudi Arabia; mrehman1@ksu.edu.sa; 2 Department of Pharmaceutical Chemistry, RAK College of Pharmaceutical Sciences, RAK Medical and Health Science University, Ras Al Khaimah 11172, UAE; 3 Department of Pharmacology and Toxicology, College of Pharmacy, Jazan University, Jazan 45142, Saudi Arabia; drandleebkhan@gmail.com; 4 Department of Pharmaceutics, College of Pharmacy, King Saud University, Riyadh 11451, Saudi Arabia; mraish@ksu.edu.sa (M.R.); mkazi@ksu.edu.sa (M.K.); usamah-14@hotmail.co.uk (O.A.); 5 Department of Clinical Pharmacy and Pharmacology, RAK College of Pharmaceutical Sciences, RAK Medical and Health Science University, Ras Al Khaimah 11172, UAE; padma@rakmhsu.ac.ae

**Keywords:** *Rhododendron arboreum* polysaccharides, lipopolysaccharide, animal model, oxidative stress, multi-biomarker approach

## Abstract

Systemic inflammation results in physiological changes, largely mediated by inflammatory cytokines. The present investigation was performed to determine the effect of *Rhododendron arboreum* (RAP) on inflammatory parameters in the animal model. The RAP (100 and 200 mg/kg) were pre-treated for animals, given orally for one week, followed by lipopolysaccharide (LPS) injection. Body temperature, burrowing, and open field behavioral changes were assessed. Biochemical parameters (AST, ALT, LDH, BIL, CK, Cr, BUN, and albumin) were done in the plasma after 6 h of LPS challenge. Oxidative stress markers SOD, CAT, and MDA were measured in different organs. Levels of inflammatory markers like tumor necrosis factor-alpha (TNF-α), interleukin-1-beta (IL-1β) and, interleukin-6 (IL-6) as well as VEGF, a specific sepsis marker in plasma, were quantified. The plasma enzymes, antioxidant markers and plasma pro-inflammatory cytokines were significantly restored (*p* < 0.5) by RAP treatment, thus preventing the multi-organ and tissue damage in LPS induced rats. The protective effect of RAP may be due to its potent antioxidant potential. Thus, RAP can prevent LPS induced oxidative stress, as well as inflammatory and multi-organ damage as reported in histopathological studies in rats when administered to the LPS treated animals. These findings indicate that RAP can benefit in the management of systemic inflammation from LPS and may have implications for a new treatment or preventive therapeutic strategies with an inflammatory component.

## 1. Introduction

Inflammation is a natural defense response against infection, toxin exposure or tissue injury [1]. Sepsis has a high mortality rate and is characterized by systemic inflammatory characteristics and organ dysfunction [2]. The occurrence of tissue injuries and organ dysfunction is critical in inflammation, hypotension, and hypoxia. A large number of agents such as eicosanoids, vasoactive oxide, and endothelins are released in sepsis, which causes vascular tone and permeability disruption [3,4,5]. Plasma leakage into the interstitial components causes edema and hypotension, negatively affecting the respiratory system and oxygenation of tissues [6]. Inflammation has become a predictable risk factor for epithelial-derived malignancies [7]. Inflammation can be triggered by autoimmune diseases, infections, tumors or any other pathological conditions that cause the body’s inflammatory cell infiltrations in the body [8]. Infiltrating cells include neutrophils, T cells, B cells, granulocytes, macrophages, and natural killing cells that excrete large quantities of inflammatory cytokines into the body. These include TNF, IL-1α, IL-1β, IL-6, IL-10 [9,10]. The cytokine balance controls the production of numerous biologically active intermediaries, which may further modify the amount of distant organ dysfunction in septic shock [10,11]. A cytokine hypothesized to regulate the extent of capillary leakage, the vascular endothelial growth factor (VEGF) plays a role in the migration of endothelial cells and proliferation and has an activity of pro coagulation [12]. VEGF has been highly potent and promotes the dose-dependent dilation of vessels than histamine which induces vascular permeability [13,14].

*R. arboreum* polysaccharides (RAP) are mainly derived from burans or gurans with potent antioxidant activities [15]. *R. arboreum* (family Ericaceae) is an evergreen shrub with a showy display of bright red flowers. It is found at altitudes as low as 1200 m and as high as 3600 m, and leaves are glossy green [16,17]. *R. arboreum* is known for its wide range and effectiveness in the treatment of eczema, diarrhea, menstrual disorders, choleretic, diuretic, antispasmodic, antioxidant, and anti-inflammatory [18,19]. As can be seen in rats, the leaves of *R. arboreum* have a hepatoprotective function [20]. The existence of alkaloids, steroids, flavonoids, tannins, and saponins has been demonstrated by phytochemical testing of *R. arboreum* [21]. In this research, it will be investigated by means of an animal model of the therapeutic effect of RAP both in systemic inflammation and on early biomarkers. In particular, we hypothesized the capacity for systemic inflammation reduction and enhances animal survival. In the present study, we also investigated the production level biochemical, oxidative, and pro-inflammatory cytokines, which are described to be crucial and responsible for the inflammatory response causing systemic inflammation.

## 2. Results

The administration of LPS causes different tissue damages, as confirmed by the significant surge in the biochemical markers. The effects of RAP on ALT, AST, LDH, BIL, CK, SCr, BUN, and albumin in plasma of control and experimental rats are shown in Table 1.

**Figure 1:**
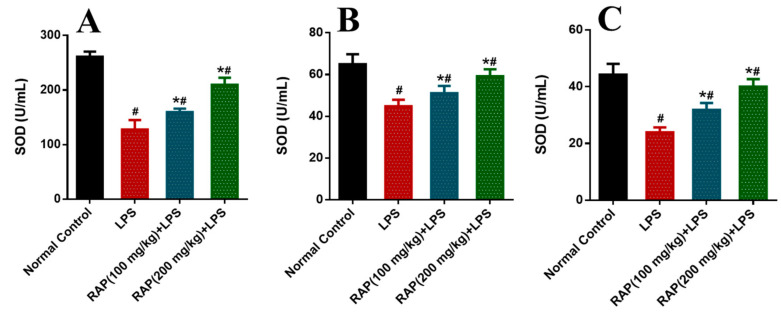
Determination of SOD in (**A**) plasma, (**B**) Liver and (**C**) Brain. Results are mean SEM of seven animals per group. ‘*’ denotes significant differences compared with the normal control group (*p* < 0.05); ‘#’ denotes significant differences compared with the LPS group (*p* < 0.05).

**Figure 2:**
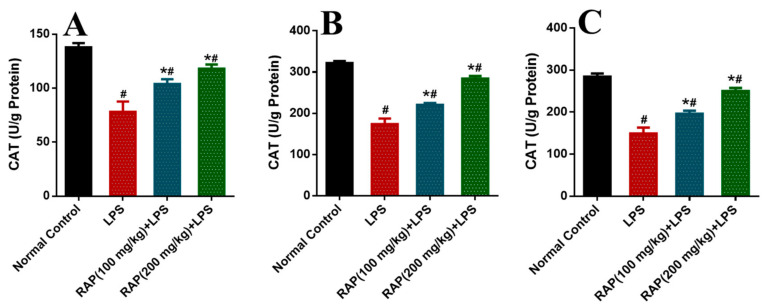
Determination of CAT in (**A**) plasma, (**B**) Liver and (**C**) Brain. Results are mean SEM of seven animals per group. ‘*’ denotes significant differences compared with the normal control group (*p* < 0.05); ‘#’ denotes significant differences compared with the LPS group (*p* < 0.05).

**Figure 3:**
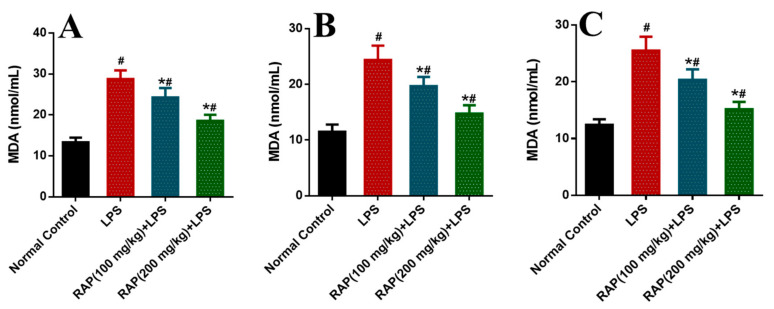
Determination of MDA in (**A**) plasma, (**B**) Liver and (**C**) Brain. Results are mean SEM of seven animals per group. ‘*’ denotes significant differences compared with the normal control group (*p* < 0.05); ‘#’ denotes significant differences compared with the LPS group (*p* < 0.05).

Significant increases were observed in the activity of these enzymes in the rat model of LPS induced inflammation compared to the normal control group. These biomarkers have been normalized by pretreatment of RAP (100 and 200 mg/kg) in a dose-dependent manner. The determination of oxidative stress markers was measured as SOD, CAT, and MDA in different tissues like plasma, liver, and brain (Figure 1, Figure 2 and Figure 3). In the balance of oxidation and the anti-oxidation mechanism, SOD plays an important role by removing superoxide anions and protecting cells against injury. Hydroxylated radical ion is the most active ROS that has strong harmful properties but can simultaneously be decomposed by CAT, MDA, and SOD. LPS injection reduces the level of SOD and CAT, while the level of MDA increases with the LPS. MDA level echoes the severity of the free radical attack. The treatment with RAP has shown a significant (*p* ≤ 0.05) change of these oxidative stress markers in a dose-dependent manner in the animals treated with LPS in all tissues.

### 2.1. Effect of RAP on Levels of TNF-α, IL-1β, IL-6, and VEGF

**Figure 4:**
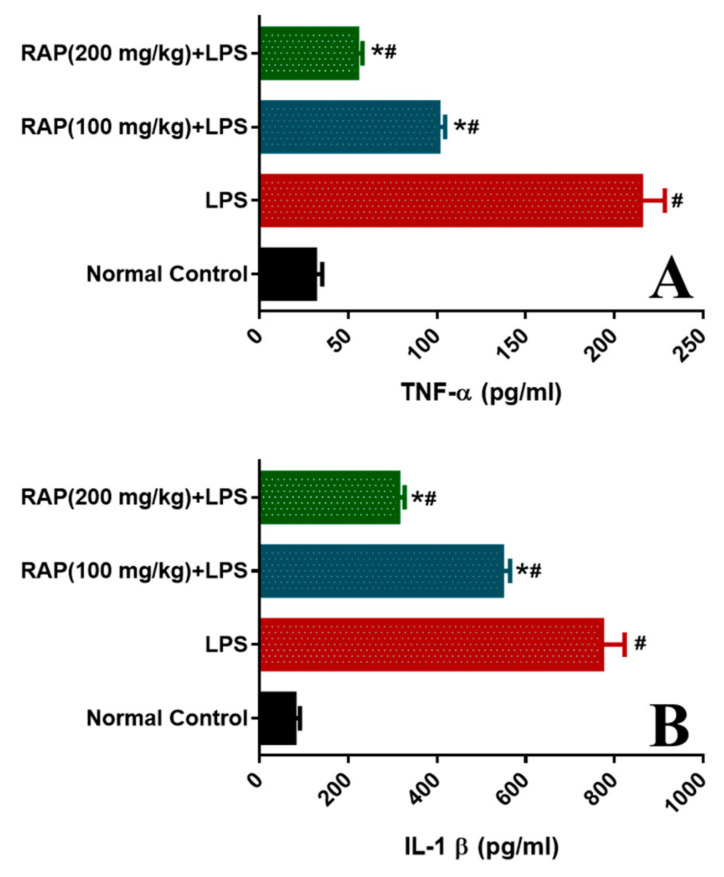
Effect of RAP (100 and 200 mg/kg) on pro-inflammatory cytokine levels in the plasma of LPS-induced systemic inflammation. (**A**) Tumor necrosis factor-a (TNF-α), and (**B**) Interleukin-1β (IL-1β). Results are mean SEM of seven animals per group. ‘*’ denotes significant differences compared with the normal control group (*p* < 0.05); ‘#’ denotes significant differences compared with the LPS group (*p* < 0.05).

To examine the modulatory influence of pretreatment of RAP (100 and 200 mg/kg) on the inflammatory cytokines and markers (TNF-α, IL-1β, IL-6, and VEGF) levels were determined. LPS administration increases the influx of pro-inflammatory cytokines. As shown in Figure 4, LPS treatment prompted a significant (*p* < 0.05) increase in TNF-α level (215.02 ± 12.14 from 31.22 ± 3.70 to pg/mL), IL-1β level (772.03 ± 45.52 from 78.19 ± 5.16 pg/mL, *p* < 0.05), and IL-6 level (1164.30 ±51.92 from 319.39 ± 16.19 pg/mL) when compared with normal control rats.

**Figure 5:**
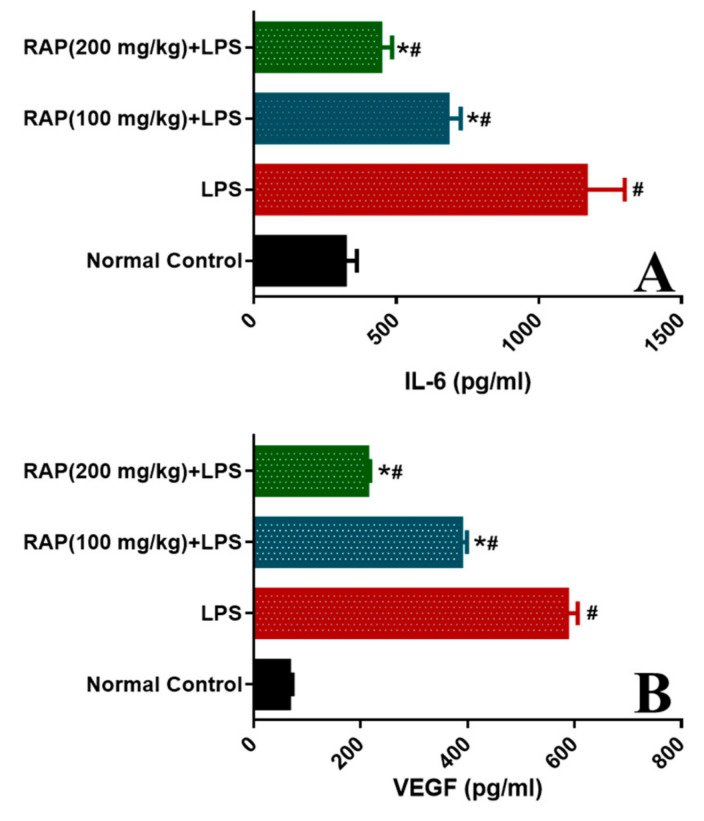
Effect of RAP (100 and 200 mg/kg) on pro-inflammatory cytokine levels in the plasma of LPS-induced systemic inflammation (**A**) Interleukin-6 (IL-6), and (**B**) Vascular endothelial growth factor (VEGF); results are mean SEM of seven animals per group. ‘*’ denotes significant differences compared with the normal control group (*p* < 0.05); ‘#’ denotes significant differences compared with the LPS group (*p* < 0.05).

Conversely, pretreatment of RAP (100 and 200 mg/kg) significantly (*p* < 0.05) reinstated the elevated levels by 53.12 and 74.45%, respectively. IL-1β levels by 29.24 and 59.50%, respectively, and IL-6 levels by 44.66 and 61.89%, respectively. These results suggest that RAP (100 and 200 mg/kg) ameliorates the upregulation of cytokines in a dose-dependent manner, and RAP exhibits potent anti-inflammatory properties. In addition, the plasma VEGF decreases with all doses by RAP (100 and 200 mg/kg), respectively (Figure 5).

### 2.2. Histopathology Results

**Figure 6:**
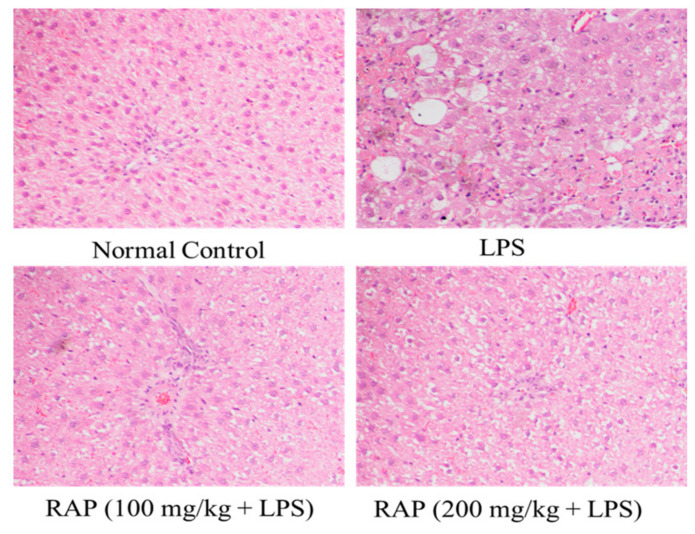
Effect of RAP (100 and 200 mg/kg) on histological alterations in the liver of LPS-induced systemic inflammation. Normal control rats showing intact hepatocytes and normal histological structure of central vein. LPS treated rats showing multiple focal necrosis, severe damage of hepatic architecture, and damage in hepatocytes. Rats treated with RAP 100 mg/kg + LPS and RAP 200 mg/kg + LPS showing absence of histopatholgical alterations.

**Figure 7:**
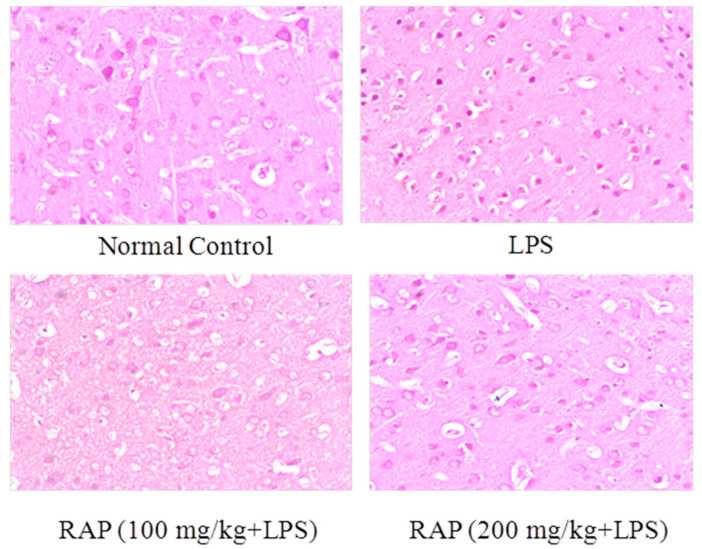
Effect of RAP (100 and 200 mg/kg) on histological alterations in the brain of LPS-induced systemic inflammation. There is a severe loss of intact cells in the cortical region of the brain. LPS group shows more vacuolated and edematous tissue in comparison to the normal control group. RAP administration has shown a decrease in neuronal loss. The normal control group has shown the normal morphology of the cortical region.

There were no architectural alterations in liver histology in sections of the liver from normal rats. LPS treated rats, however, showed cell infiltration, necrosis, sinusoidal dilatation, and severe congestion of the central vein, while 100 and 200 mg of RAP/kg suppresses these LPS induced histopathological changes (Figure 6). Histological alterations have been reported in the LPS group in the brain. The cells with pyknotic nuclei are regarded as dead. Pyknotic nuclei (darkly stained punctate nuclei) and altered vacuolated morphology were observed in the LPS group. RAP treatments (100 and 200 mg/kg) have successfully reduced the injury and restored the normal brain architecture (Figure 7).

## 3. Discussion

This study was designed to investigate the effects of previously identified RAP polysaccharides [15] on some of the biochemical, antioxidant, inflammatory biomarkers, and histological alterations in an animal model of inflammation. The results indicate that oral administrations of RAP with doses of 100 and 200 mg/kg before LPS administration reduced the levels of total inflammatory cytokines and markers. In the therapeutic approaches for organ injury after LPS administration, additional antioxidant compounds capable of preventing this damage is necessary. This research sheds light on the therapeutic efficacy of RAP extract against the systemic inflammatory response induced by LPS in rats. The current results showed that LPS administration encouraged an increase in plasma AST, ALT, and LDH activity, which could be due to an increase in hepatic cell membrane fluidity that led to the rerelease of the enzymes into the circulation. Pretreatment with RAP (100 and 200 mg/kg) significantly reduces the plasma concentration of AST, ALT, and LDH in inflammatory animals, which indicates the maintenance of hepatic cell function and structure. Such an increase in enzyme activity may be due to the antioxidant properties of the RAP to free radicals, thus protecting the integrity of the cell membranes from oxidative damage caused by LPS. Results of the current investigation showed that after administration of LPS, total bilirubin in inflammatory rats increased significantly. LPS administration causes several changes that lead to intrahepatic cholestasis-related hyperbilirubinemia, the severity of which depends on the degree of impairment of bile formation [22,23,24]. RAP (100 and 200 mg/kg) pretreatment resulted in a significant decrease in BIL in a dose-dependent manner. Following LPS administration, this study found a significant increase in CK, Cr, and BUN. These findings are consistent with the findings of Wali et al., 2020 [25]. The results obtained indicate that, compared to normal groups, oral administration of RAP significantly reduced CK, Cr, and BUN activities. The loss of renal function is associated with an organ failure, which leads to the accumulation of a number of compounds during kidney disease development [26]. In this study, changes in tissues and organs caused by oxidative stress were biochemically examined through experimentally inflicted systemic inflammation in rats by LPS. SOD and CAT are enzymes; SOD catalyzes hydrogen peroxide and oxygen degradation of superoxide, while CAT catalyzes the reduction of hydrogen peroxide reaction and thus prevents oxidative damage in cells [27]. These act equally as supportive antioxidant enzymes that offer protection against ROS [28,29]. This study showed significant reductions in the liver and brains of LPS treated animals in these enzymes. In the present study, however, treatment with RAP (100 and 200 mg/kg) restored these activities. The present results observed a significant reduction in the albumin concentration following LPS administration. The decrease in albumin levels in the current investigation might be due to severe damage to the liver leading to a reduction in the albumin synthesizing capacity [26,29]. Pretreatment with RAP has restored the albumin concentration in a dose-dependent manner.

The life-threatening organ injury that occurs during gram-negative sepsis is not primarily the result of direct injurious actions of the bacteria but is postulated to be due to an excess of endogenous factors released by the host in response to the LPS portion of the gram-negative cell wall [30]. TNF-α is an endogenous mediator associated with the initiation of events as a major proximal factor, which ultimately leads to organ injury and death in both animals and humans due to gram-negative sepsis. A number of experimental evidence suggests that TNF-α is a pivotal player in gram-negative sepsis pathogenesis. First, purified, endotoxin-free recombinant TNF-α induces the same clinical and pathological changes in experimental animals, as seen in gram-negative sepsis in patients [31]. Second, elevated circulating levels of TNF-α occur in patients with septic shock, and levels correlate with clinical outcomes [32]. Third, neutralizing antibodies to TNF-α protect against the lethality of either LPS or gram-negative bacteremia in various animal models [31,33]. This evidence suggests that, when used appropriately, antagonists of TNF-α production or actions may be effective in treating septic shock [31,32,34]. To treat gram-negative sepsis effectively with therapeutic strategies directed against potentially injurious endogenous factors, it is imperative to recognize the temporal expression of these endogenous factors in vivo. For example, dexamethasone is a potent inhibitor of LPS-stimulated TNF-α production, suppressing TNF-α synthesis and release both at the transcriptional and post-transcriptional levels [35]. However, dexamethasone has proved to be poorly effective in treating septic shock in clinical studies [35,36]. This difference in effect is at least partially associated with the rapid temporal expression of TNF-α and the time of administration of dexamethasone. Dexamethasone is an effective inhibitor of TNF-α production if administered shortly before or after LPS challenge but is much less effective in inhibiting TNF-α production when administered 20 min after LPS [37]. One reason why dexamethasone may be ineffective in the treatment of some clinical cases of septic shock is that it is administered too long after the initiating stimulus. Similarly, neutralizing antibodies against TNF-α is only effective in inhibiting the lethal effects of LPS or gram-negative bacteria when administered before or in some models shortly after the stimulus [37]. In the present study, our data demonstrated that pretreatment of RAP (100 and 200 mg/kg) has potential to inhibit LPS induced inflammation as evident by the decreased plasma TNF-α circulating levels.

Cytokines are immune response regulators for infection and play an important role in inflammation and trauma management [38]. IL-1β is a member of the interleukin-1 family of cytokines, also known as catabolin [38]. IL-1β produces as a protein through activated macrophages, which are processed in a proteolytic fashion by caspase-1. In inflammatory reactions, IL-1β is an important mediator of various cellular activities, such as cell differentiation, proliferation, and apoptosis, proposing that IL-1β plays a key role in microbial infections [38,39]. IL-6 is a pleiotropic interleukin that works as a pro and anti-inflammatory cytokine [10]. T-cells and macrophages secrete IL-6, which triggers an immune reaction to trauma and other inflammatory tissue injuries, in response to a specific microbial molecule, called pathogen-associated molecular patterns (PAMPs) [10]. These PAMPs bind to a main class of molecules called pattern recognition receptors, such as toll-like receptors (TLRs) of the innate immune system. They appear on the outside and intracellular compartments and cause the production of cytokines. Therefore, IL-6 appears in several disease processes such as cardiovascular, autoimmune, cancer and in septic disorders [40,41,42]. The results of the present study indicate that pretreatment of RAP (100 and 200 mg/kg) to LPS treated rats showed significant reduction in the circulating plasma levels of IL-1β and IL-6 as compared to the LPS alone treated animals. These results are in agreement with the previous finding of Wali and co-workers [25], which indicated that IL-1β and IL-6 blockade may be an important novel treatment strategy against overwhelming sepsis or septic shock.

VEGF, which is the most potent angiogenic growth factor and pro-inflammatory cytokine in endothelial cells, stimulates migration, proliferation, and tube formation [43]. In fact, VEGF increases leucocyte infiltration through adhesion protein ICAM-1, which is necessary to induce or increase inflammation by the interaction of leukocytes [44,45,46]. Various factors are involved in the regulation of VEGF, and consequently, they influence angiogenesis. Many inflammatory mediators, especially PGE2, NO, TNF-α, and IL-1, can stimulate both the VEGF mRNA and protein levels [47,48]. VEGF circulating levels are considerably increased in the rat plasma and have been observed in the current results. Similar findings have been reported in human studies where VEGF levels have been shown to be increased in the plasma with sepsis [49,50]. The present results have also demonstrated that VEGF is upregulated in LPS induced systemic inflammation. Inhibition of VEGF with RAP (100 and 200 mg/kg) pretreatment also attenuates TNF-α and IL-1β during inflammation. This data proposes a role for VEGF in regulating inflammatory cytokine production in LPS induced inflammation. The outcome of augmented levels of VEGF in the present study of animal inflammation proposes a potentially essential role in the host inflammatory response. The study shows that RAP improves systemic inflammation of LPS by restoring biochemical and antioxidant indices. The histopathological findings are consistent with the current results that pretreatment with RAP (100 and 200 mg/kg) protects the hepatic and brain architecture in LPS-treated animals.

## 4. Materials and Methods

### 4.1. Extraction of Polysaccharides

The *R arboreum* polysaccharides used in the current study were extracted as previously reported by Ahmad et al., [15].

### 4.2. Drugs and Chemicals

Lipopolysaccharide (LPS) was purchased from Sigma-Aldrich (St. Louis, MO, USA). All pro-inflammatory and inflammatory rat enzyme-linked immunosorbent assay (ELISA) kits were bought from (My-BioSource, CA, USA, and BioVision, Inc. Milpitas, CA, USA). All other chemicals used were of good quality.

### 4.3. Animals and Study Protocol

Wistar rats (Male: 195–205 g) six-week-old were acquired from the animal house and were housed in plastic cages. At a room temperature of 25 °C, 10% of humidity, and 12/12 light-dark cycle. The rats have been given free access to water and food. The study protocol (UAEU-EC-11-2020-FP) was approved by ethics committee. The rats were divided into four groups with (*n* = 6) in each group. Animals in Group 1 received normal saline and served as normal control. LPS (10 mg/kg i.p.) were given to animals in Group II and served as disease control [25]. Animals in Group III and IV were given RAP 100 and 200 mg/kg p.o., respectively, for seven days, and LPS was given 2 h after the last doses of RAP. The animals were sacrificed under anesthesia after 6 h of LPS treatment, and blood samples were placed in sterile heparin tubes for plasma separation. For biochemical and inflammatory marker analysis, the plasma samples were kept in −80 °C. The liver and brain were also collected to study the histopathological alterations.

Colorimetric methods were applied for the evaluation of biochemical parameters, including aspartate aminotransferase (AST), alanine aminotransferase (ALT), lactate dehydrogenase (LDH), bilirubin (BIL), creatine kinase (CK), creatine (Cr), blood urea nitrogen (BUN), and albumin (Human Diagnostic Worldwide; Wiesbaden, Germany). Antioxidant enzyme indices were measured in plasma, brain, and liver. The liver and brain tissues were collected and homogenized based on the requirements for superoxide dismutase (SOD), malondialdehyde (MDA), and catalase (CAT) kits and were determined according to the manufacturer guidelines.

Inflammatory markers TNF-α, IL-1β, and IL-6 and sepsis biomarker vascular endothelial growth factor (VEGF) were determined by using an ELISA technology as per the directions of the manufacturer (Booster Biological Technology Co., Ltd., Pleasanton, CA, USA).

The liver and brain were removed after perfusion of animals and were further fixed for 48 h in 4% paraformaldehyde in 10 mM PBS (pH 7.4). The tissues are embedded with paraffin, and coronal sections of 5 µM thickness were cut for staining of hematoxylin and eosin (H & E). The sections were examined under the microscope for any abnormality and the dead cells in each group.

### 4.4. Statistical Analysis

All results have been shown as mean ± SEM. Differences among groups were statistically significantly determined by means of a one-way variance analysis (ANOVA) followed by multiple comparisons. *p*-value ≤ 0.05 were considered significant.

## 5. Conclusions

The present study demonstrated the inhibitory effects of RAP on the animal model of inflammation. Here, we showed that RAP significantly restores the biochemical enzymes, antioxidant defense, reducing oxidative stress, inflammatory markers (TNF-α, IL-1β, IL-6, and VEGF) levels, and restores liver and brain injuries triggered by LPS. Moreover, the histopathological interpretations showed that LPS induced hepatic and brain injuries were greatly improved by RAP pretreatment in a dose-dependent manner. The present results suggest that RAP provides protection against inflammation-induced tissue and organ injury via regulating oxidative and inflammatory responses. RAP could be used to inhibit or diminish LPS induced inflammatory injury.

## 6. Limitations and Future Directions

*Rhododendron arboreum* possess good quantities of flavonoids, polyphenolic compounds and polysaccharides. The present study determined the total phenolics (TPC) as well as total flavonoid contents (TFC) but these values were found to be very insignificant (TPC < 10 mg GAE/g of extract and TFC < 20 mg RE/g of extract) and hence were not reported. This decline in TFC and TPC content may be due to the climatic change or habitat of the plant. Very low proportions of TFC and TPC support the results indicating polysaccharides as most abundant phytoconstituents present in the extract. Moreover, the polysaccharides from *R. arboreum* have already been proved for their anti-inflammatory potential. Polysaccharide research poses a range of obstacles which must be addressed. Current research on polysaccharides will solve the ambiguities related to their therapeutic effectiveness and clinical trials. To fill this knowledge gap, the medicinal value of polysaccharides for inflammatory diseases have been presented to determine the popularity and usage. As per our knowledge this is the first report on role of RAP in ameliorate pathogen induced inflammatory disorder. Moreover, reliable and cost-effective methods for modifying, processing and full identification of the ingredients of the extracts will further provide the actual insights about the compounds responsible for biological activity, and a substantial area of research has therefore yet to be explored.

## Figures and Tables

**Table 1:** Biochemical assessment between normal and inflammatory rats treated with different dose of RAP.

Parameters	Normal Control	LPS 10 mg/kg	RAP 100 mg/kg	RAP200 mg/kg
AST (U/L)	52.19 ± 2.99	237.88 ± 9.86 *	157.66 ± 6.31 *^,#^	71.83 ± 4.19 *^,#^
ALT (U/L)	39.41 ± 2.39	215.25 ± 6.91 *	106.37 ± 4.59 *^,#^	72.36 ± 3.69 *^,#^
LDH (U/L)	305.32 ± 9.56	1026.33 ± 31.65 *	786.25 ± 22.15 *^,#^	562.69 ± 19.85 *^,#^
BIL (µmol/L)	1.55 ± 0.42	7.86 ± 1.11 *	5.44 ± 0.95 *^,#^	2.86 ± 0.81 *^,#^
CK (U/L)	72.88 ± 4.96	989.52 ± 20.96 *	731.43 ± 17.96 *^,#^	621.22 ± 21.39 *^,#^
Cr (mg/dL)	0.37 ± 0.05	0.66 ± 0.03 *	0.50 ± 0.04 *^,#^	0.42 ± 0.03 *^,#^
BUN (mg/dL)	52.23 ± 1.84	155.46 ± 6.44 *	95.43 ± 4.83 *^,#^	71.92 ± 3.61 *^,#^
Albumin (g/dL)	3.92 ± 0.36	2.49 ± 0.31 *	2.86 ± 0.39 *^,#^	3.45 ± 0.28 *^,#^

‘*’ denotes significant differences compared with the normal control group (*p* < 0.05); ‘^#^’ denotes significant differences compared with the LPS group (*p* < 0.05).
